# mCME project V.2.0: randomised controlled trial of a revised SMS-based continuing medical education intervention among HIV clinicians in Vietnam

**DOI:** 10.1136/bmjgh-2017-000632

**Published:** 2018-02-26

**Authors:** Christopher J Gill, Ngoc Bao Le, Nafisa Halim, Cao Thi Hue Chi, Viet Ha Nguyen, Rachael Bonawitz, Pham Vu Hoang, Hoang Long Nguyen, Phan Thi Thu Huong, Anna Larson Williams, Ngoc Anh Le, Lora Sabin

**Affiliations:** 1 Department of Global Health, Boston University School of Public Health, Boston, Massachusetts, USA; 2 Consulting Research for Community Development, Hanoi, Vietnam; 3 Vietnam Authority for AIDS Control, Ministry of Health, Hanoi, Vietnam; 4 Center for Population Research Information and Databases (CPRID), General Office for Population and Family Planning, Ministry of Health, Hanoi, Vietnam; 5 Hanoi Medical University, Hanoi, Vietnam

**Keywords:** health policy, health education and promotion, public health, randomised control trial

## Abstract

**Background:**

Continuing medical education (CME) is indispensable, but costs are a barrier. We tested the effectiveness of a novel mHealth intervention (mCME V.2.0) promoting CME among Vietnamese HIV clinicians.

**Methods:**

We enrolled HIV clinicians from three provinces near Hanoi. The 6-month intervention consisted of (1) daily short message service multiple-choice quiz questions, (2) daily linked readings, (3) links to online CME courses and (4) feedback messages describing the performance of the participant relative to the group. Control participants had equal access to the online CME courses. Our primary endpoint was utilisation of the online CME courses; secondary endpoints were self-study behaviour, performance on a standardised medical exam and job satisfaction.

**Results:**

From 121 total HIV clinicians in the three provinces, 106 (87.6%) enrolled, and 48/53 intervention (90%) and 47/53 control (89%) participants completed the endline evaluations. Compared with controls, intervention participants were more likely to use the CME courses (risk ratio (RR) 2.3, 95% CI 1.4 to 3.8, accounting for 83% of course use (P<0.001)). Intervention participants increased self-study behaviours over controls in terms of use of medical textbooks (P<0.01), consulting with colleagues (P<0.01), searching on the internet (P<0.001), using specialist websites (P=0.02), consulting the Vietnam HIV/AIDS treatment guidelines (P=0.02) and searching the scientific literature (P=0.09). Intervention participants outperformed controls on the exam (+23% vs +12% score gains, P=0.05) and had higher job satisfaction.

**Conclusion:**

The mCME V.2.0 intervention improved self-study behaviour, medical knowledge and job satisfaction. This approach has potential for expansion in Vietnam and similar settings.

**Trial registration number:**

NCT02381743.

**Key questions**

**What is already known about this topic?**

- It is essential that clinicians participate in continuing medical education (CME) in order to retain/update their skills and knowledge.
- The high costs and inconvenience of in-person CME workshops is a significant challenge, particularly in low-income and middle-income countries.
- Little is known about whether text messages could encourage engagement and achievement in CME, as an alternative to in-person CME.
- Prior mHealth educational studies have either failed to find any impact on medical learning or were focused around very narrow areas of medical knowledge/practice.
- Our intervention may be the first to test a short message service-based mHealth educational intervention that aims to improve knowledge across a broad range of clinical practices.

**What are the new findings?**

- Our novel mobile continuing medical education (mCME) intervention was simple to implement, well accepted and had consistently high participation rates.
- mCME led to significant increases in self-study behaviours across a broad range of objectively and subjectively measured domains.
- mCME increased medical knowledge on a standardised medical examination.
- mCME had the secondary benefit of improving job satisfaction.

**Recommendations for policy**

- mCME has high potential as a pedagogical tool for promoting CME via distance learning and may be particularly useful in countries with limited resources for traditional, in-person CME activities.

## Introduction

In the USA and many other high-income countries, clinicians are required to maintain clinical knowledge through participation in accredited continuing medical education (CME) activities. Increasingly, these are delivered via internet-based, self-educational modules, such as clinical vignettes accompanied by multiple-choice questions. Such CME courses are principally offered over the internet for access using desktop or laptop computers. However, this approach could be adapted for mobile phones using short message service (SMS) text messaging instead, a significant advantage in settings where cell phone ownership is ubiquitous but access to computers is limited.

As with many countries, the government of Vietnam is seeking to develop strategies to maintain the skills of its medical workforce. In November 2009, Vietnam passed the ‘Law on Medical Examination and Treatment’ mandating that all clinicians be licensed and that CME be required to sustain licensure. Given limited resources, our partners in the Ministry of Health settled on distance learning as the most efficient solution for implementing this mandate. Uncertainty about the best way to achieve high uptake and impact created an opportunity to test innovative strategies for distance learning. In this context, we have partnered with Vietnam’s Ministry of Health (MOH) to generate robust evidence around a novel, mobile phone-based approach for promoting CME via SMS text messages.

From 2014 to 2015, we conducted a National Institutes of Health (NIH)-supported randomised controlled trial (RCT) of an SMS-based distance learning intervention: the mobile continuing medical education (mCME) project.^1^ In that initial study, which we refer to as mCME V.1.0, 638 Vietnamese community-based physician’s assistants (CBPAs), a mid-level cadre of healthcare workers who provide the majority of primary care services nationwide, were randomised to either a comparison group or one of two mCME intervention groups that received either (1) daily text messages presenting medical facts or (2) daily text messages on these same topics but phrased as multiple-choice questions. Unfortunately, while the intervention proved to be well accepted, technically feasible and inexpensive, it failed to achieve our primary objective, which was to improve medical knowledge.

**Figure 1:**
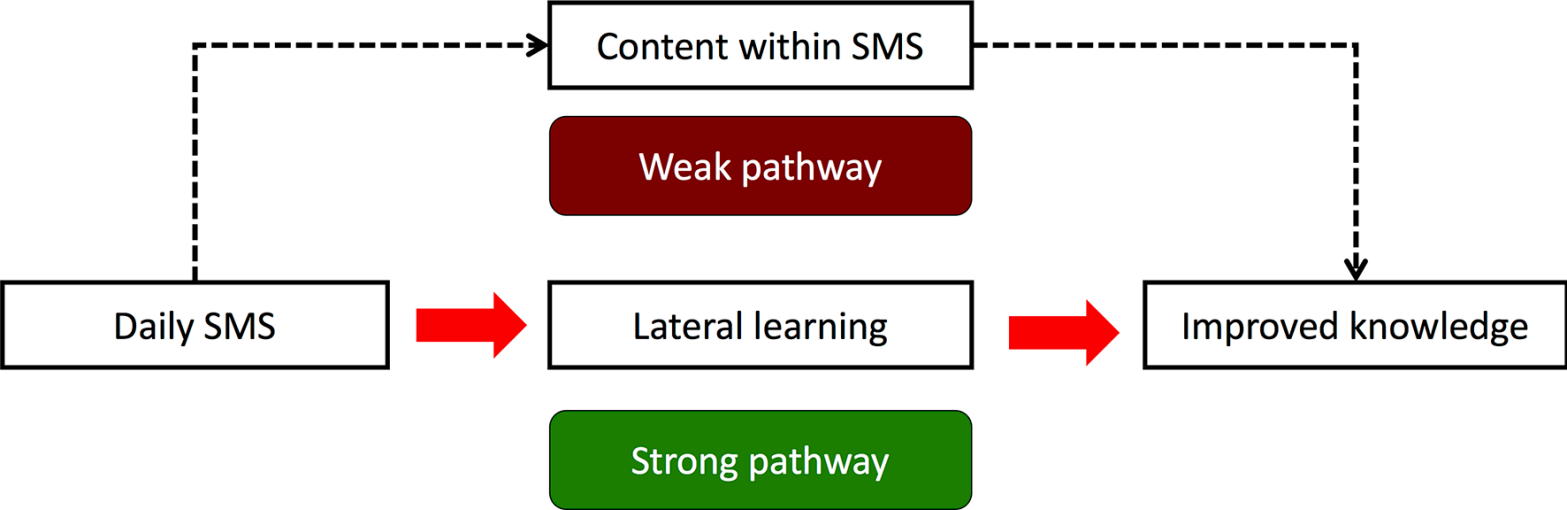
Conceptual model for how the mobile continuing medical education (CME) intervention might improve medical knowledge. In this schema, we hypothesise that there is a weak and a strong pathway leading to improved knowledge. The weak pathway is driven by the content within the short message service (SMS) messages themselves, which are necessarily brief and hence limited in volume of information they can provide. The strong pathway assumes instead that the SMS messages are important primarily as a cue to action that encourage self-study on the topics presented in the messages. This means going beyond the message to explore related content on that topic, which could be via use of the linked readings or the CME courses in the intervention. But this also encompasses any of many appropriate strategies for self-study, as preferred by each participant. We refer to this process of studying beyond the SMS messages as ‘lateral learning’.

As represented in our conceptual model (figure 1), given the brevity of text messages (≤160 characters) we view mCME more as a behavioural change intervention for promoting self-study behaviours than as a platform for conveying complex medical information per se. Hence, we depict the content in the messages themselves as the ‘weak pathway’. By contrast, we hypothesised that SMS messages could be quite potent as ‘cues to action’ as theorised by the Health Belief Model,^2 3^ with the messages serving as a pedagogical tool that direct students towards relevant thematic areas and motivates effective self-study on those topics. The desired result is that students will go beyond the daily quizzes to learn about the topics in greater depth, a process we term ‘lateral learning’. This is represented by the ‘strong pathway’.

Unfortunately, in mCME V.1.0, participants did not engage the strong pathway. Rather, they accepted SMS messages quite literally, and many prepared for the exam by studying the answers to the text messages.^1 4^ Participants did not increase the overall amount of time they spent on weekly self-study and engaged in ineffective activities for seeking answers to daily questions, such as Google searches, or asking fellow CBPAs for their opinions. While participants generally reported having access to medical textbooks, their use of formal medical texts was low at baseline and did not increase.

Rather than abandoning the mCME concept, we were encouraged by the enthusiasm and high participation rates during the first RCT, and our partners in Vietnam’s MOH saw potential in the intervention if it could be suitably improved. Based on feedback from the participants and discussions with our partners, we modified the intervention in key areas to better promote lateral learning, creating mCME V.2.0 (see table 1).^1 4^ The updated intervention provided more structure around course content, and, by providing performance feedback SMS messages, strengthened the game-like aspects of the intervention. Here we report the results from this second NIH-supported RCT, conducted during 2016–2017 among Vietnamese HIV clinicians in three provinces. We assessed whether mCME V.2.0 improved clinicians’ self-study behaviours, whether it improved their medical knowledge and whether it had a positive impact on their reported job satisfaction.

**Table 1:** Design modifications incorporated into the mobile continuing medical education (mCME) V.2.0 intervention

Limitations of mCME V.1.0	Changes incorporated into mCME V.2.0
1. Daily quiz content did not build sequentially.	Content bundled into 1–3 week-long modules around a single theme (eg, HIV and tuberculosis)
2. Intervention did not provide links to technical information related to the daily quiz questions	SMS messages included hyperlinks to technical readings aligned with the daily quiz question
3. SMS messages were not integrated with other distance learning modalities	Each module began and ended with SMS messages including hyperlinks to online CME course on that same topic
4. Motivational feedback would make the SMS quizzes more engaging	At the completion of each module, participants received an SMS summarising their performance on the module’s quizzes along with the group’s average performance
SMS, short message service.	

## Methods

### Study overview

Our key implementing partner and collaborator was the Vietnamese non-governmental organisation Consulting, Researching on Community Development. Our key MOH partners were the Center for Population Research Information and Databases, a subdivision of the General Office for Population and Family Planning, whose information technology (IT) group developed the SMS software for mCME V.1.0 and V.2.0 and whose servers hosted the intervention; and the Vietnam Authority for AIDS Control. Our academic partner was Hanoi Medical University (HMU). The study was registered on clinicaltrials.gov as NCT02381743. The trial protocol is provided in online supplement 1, protocol. In mCME V.1.0, our target user population were CBPAs working in primary care. Funding for mCME V.2.0 came through a supplemental NIH award that required a focus on HIV/AIDS. To comply with this requirement, for mCME V.2.0, we switched our participant population from CBPAs to HIV clinicians.

10.1136/bmjgh-2017-000632.supp1 Supplementary file 1

### Enrolment and baseline procedures

All participants provided written informed consent. The participants in the current study were HIV clinicians working in three provinces in Northern Vietnam: Thái Nguyên, Hải Phòng and Quảng Ninh. Enrolment occurred at three workshops, one per participating province; all clinicians who provided services to HIV-infected patients were invited to participate. Inclusion criteria were (1) owning a smartphone, (2) being certified to provide HIV/AIDS care, (3) willingness to adhere to study procedures and (4) providing informed consent.

At the baseline workshops, enrolled participants were randomised in a 1:1 ratio using a SAS Macro to control or intervention groups in blocks of 4.

Participants were then introduced to the online HMU CME courses. Prior to the study, we had selected 15 courses that were deemed by consensus of the investigators to be of highest priority to HIV clinicians (table 2). Each course consisted of a suite of documents including at least one video lecture, one or more readings and an end of module quiz (distinct from our daily SMS quizzes). All participants were instructed how to use the HMU courses, and provided unique log-in ID numbers (that corresponded to their study ID numbers) and passwords, and instructed that the range of topics in the online courses would be represented by the modules during the intervention and would be reassessed in a second endline examination in 6 months. Participants in both groups were encouraged to take full advantage of the HMU courses and could access the materials at any time, and as often as they chose. Participants were informed that their use of these courses would be monitored on an individual basis when participants logged in using their study ID numbers and clicked through the web pages contained in each course.

**Table 2:** HIV/AIDS topic areas addressed in mobile continuing medical education V.2.0

Module number	Thematic areas covered in module
1	HIV counselling and testing
2	HIV in children
3	HIV in adults
4	Antiretroviral therapy in pregnancy
5	Antiretroviral drug resistance
6	Managing treatment failure
7	Diarrhoeal diseases in HIV/AIDS
8	HIV and tuberculosis coinfections
9	HIV and viral hepatitis
10	Common skin problems in HIV/AIDS
11	*Penicillium marneffei* infections
12	HIV-associated lymphomas
13	Long-term effects of HIV and antiretroviral therapy
14	Neurocognitive problems in HIV
15	Palliative care and methadone replacement therapy

Modules lasted 1–3 weeks, and therefore included between 7 and 21 daily topics, in addition to the introductory and concluding SMS and the feedback SMS sent the day after completing a module. Modules ran on a Monday to Sunday schedule.

SMS, short message service.

Following this, all participants provided baseline demographic data, completed a job satisfaction survey (the Brief Index of Affective Job Satisfaction)^5^ and then took a 100-item standardised, multiple-choice test of medical knowledge focused on aspects of HIV care covered in the 15 HMU courses. Categorical data from participants, including responses to job satisfaction and exam responses, were collected at baseline and endline using bubble sheets, and scored using an optical scanner.

The randomisation list, linked to study ID numbers, was in turn linked to specific versions of the examinations to be taken at baseline and endline, and generated in advance by the study statistician. As described previously, this was intended to balance the difficulties of the baseline and endline examinations and to minimise sharing of answers during the examinations themselves.^1^

### Intervention procedures

The intervention commenced shortly after all baseline workshops were completed, with weekly (controls) or daily (intervention) SMS messages being delivered daily for the next six months.

**Figure 2:**
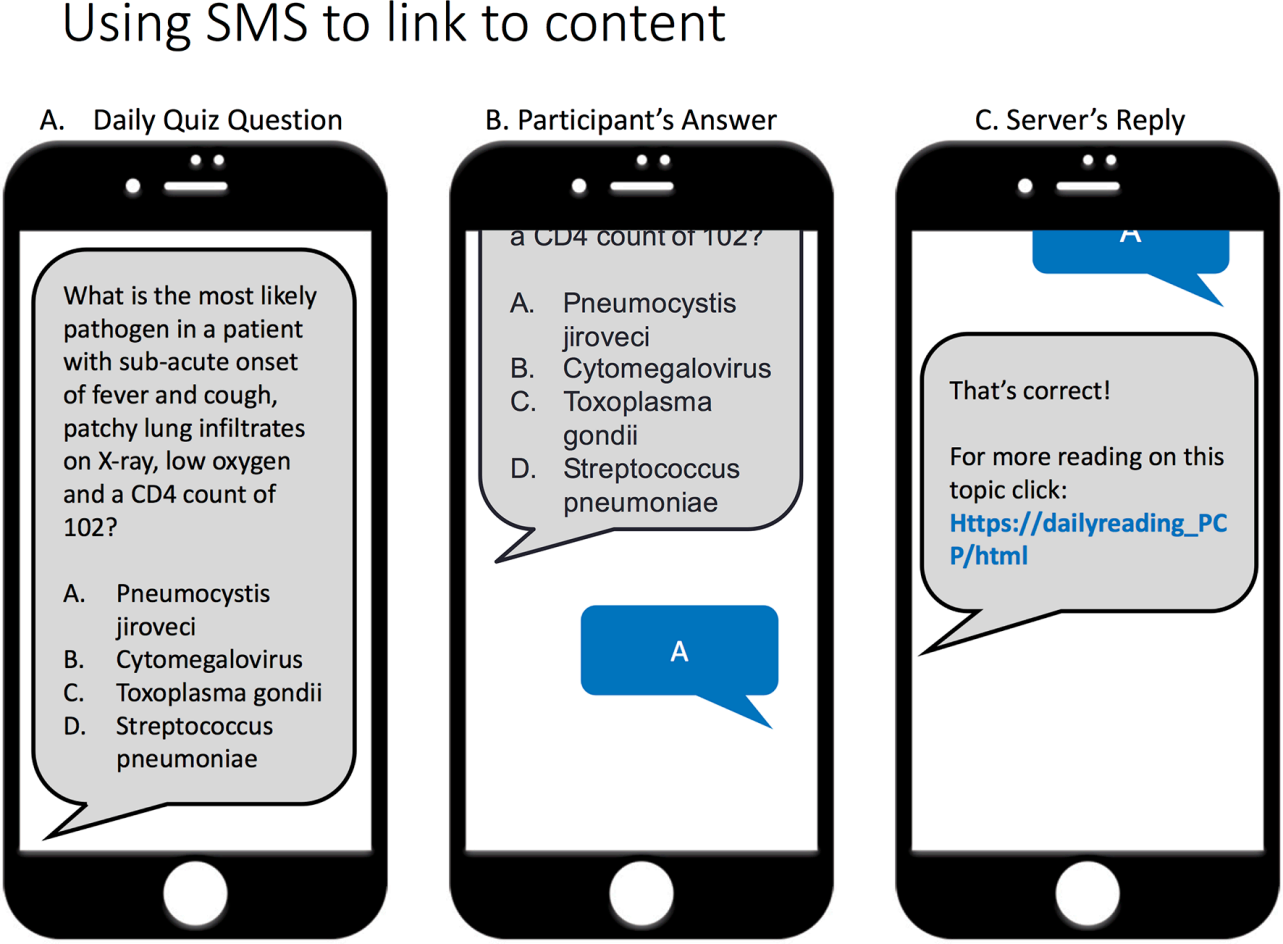
Example of a hypothetical daily short message service (SMS) quiz sequence The cartoon depicts the sequence for a daily SMS quiz question. An intervention participant receives a four-item multiple choice question (A), keys in their response (B) and then receives an automatically generated reply from the server congratulating for correct answers or encouraging better luck next time by providing the correct answer (C). In all cases, the final message contains a hyperlink to further technical reading on that same topic, typically 1–3 paragraphs in length. In most cases, these readings were based on the Vietnam HIV/AIDS Treatment Guidelines 2015 handbook.

The sequence of SMS messages to intervention participants was as follows:

1. At the start of each module, participants received a welcome message that included a hyperlink to the HMU course on that topic.
2. Participants then received their first SMS quiz question, presented as a four-item multiple-choice question. Participants keyed in an answer (‘A’ or ‘a’, ‘B’ or ‘b’, ‘C’ or ‘c’, ‘D’ or ‘d’); all other answers would register as ‘incorrect’ in the system.
3. The system responded with an SMS indicating if their answer was correct and included a hyperlink to a daily technical reading on the same topic (figure 2). This cycle was repeated each day for the remainder of the module.
4. On the final day or each module, after completing the last question, participants received a message congratulating them on completing the module and again provided a hyperlink to the HMU CME course on that topic.
5. The morning after a module ended intervention participants also received a message summarising their performance on the daily quizzes for the prior module along with the group’s average score.

This sequence was repeated through all 15 modules of the series over a total of 6 months.

Control participants received a weekly non-medical SMS, merely to remind them that they were part of a study, but did not receive daily quizzes, links to daily readings, SMS invitations to the HMU courses or performance feedback SMS messages. We selected weekly rather than daily SMS messages for the control participants for two reasons: 1) to reduce costs; and 2) out of concern that even daily non-medical messages might prove to be a stimulus to behavioural change, which would make it more difficult to observe the effect of the intervention itself.

### Endline procedures

At endline, all participants completed a second survey focused on study behaviours, repeated the job satisfaction survey and took the endline medical examination. Subgroups of participants with either high or low SMS quiz participation rates were then approached to join focus group discussions in a set of qualitative analyses, whose results will be presented separately.

### Analytic methods

The power calculations around our primary endpoint, the use of the online HMU courses, was constrained by our resources for the supplemental award that funded mCME V.2.0. This constraint meant that we could only afford to work in three provinces, and each province only had a finite number of HIV clinicians. There were ~120 HIV clinicians in the three provinces and we aimed to enrol all of them, but were ultimately only able to enrol 106. Hence, while the study objectives were defined a priori, given no prior knowledge of the effect size of daily SMS messages on our primary endpoint (CME course utilisation), our power calculations are provided merely to describe a range of scenarios under different effect sizes and a cohort size of 106. Therefore, using an uncorrected χ^2^ test, with 53 persons per group, this number meant that we would be able to detect, with an alpha of 0.05, a difference of 30% in course utilisation with 91% power, a 25% difference with 77% power and a 20% difference with 56% power.

For our primary endpoint, use of the HMU courses, analysis was by intention to treat (ITT) since the outcome was measured longitudinally and included all randomised participants. For the secondary endpoints based on data only collected at the endline assessment (study behaviours, job satisfaction), data were limited to participants who attended the endline workshop and were therefore per-protocol analyses. For the analysis of change in medical knowledge on the standardised examination from baseline to endline, we also conducted an ITT analysis in which we imputed endline exam results for the participants who did not attend the endline workshop by carrying their baseline exam scores forward (ie, assuming, conservatively, that there was no improvement in exam performance).

Outcomes were compared using tests of proportions, paired t-tests or analysis of variance as required. Non-parametric tests were used to contrast dichotomous or multilevel responses, such as time engaged in medical self-education. For the primary endpoint, we compared the total utilisation of HMU courses by intervention versus control participants, as well as the probability as a relative risk that an intervention subject versus control would ever use the HMU courses, along with 95% CIs. All analyses used SAS V.9.4, or Microsoft Excel.

## Results

### Study disposition

Invitations to participate were sent to all HIV clinicians in the three provinces (n=121). The 106 who enrolled accounted for 87.6% of all clinicians who could have enrolled, making this a highly representative sample. Participant baseline characteristics are shown in table 3 and were well balanced between the groups. On average, clinicians were about 40 years of age, had worked in HIV/AIDS for about 4 years and were slightly more likely to be women. Roughly 40% were MDs, with the rest holding mid-level provider certifications. All had completed official government-sponsored HIV/AIDS training leading to specialist certification. They tended to work in groups of 1–7 clinicians and saw 0–9 patients per day. Most reported 1–2 hours spent on medical self-study per week at baseline.

**Table 3:** Baseline participant characteristics

	Intervention (n=53)	Control (n=53)
Age (years)	41.5	40.8
Years worked as clinician focused on HIV/AIDS	4.3	4.2
Male (%)	42	47
Female (%)	58	53
Clinical training		
Mid-level provider (%)	60	55
MD (%)	40	45
Patients seen per day (n)		
0–9 (%)	51	66
10–19 (%)	13	11
20–29 (%)	15	8
30–39 (%)	8	8
40+(%)	13	8
Clinicians in same practice (n)		
1–2 (%)	21	41
3–4 (%)	45	22
5–7 (%)	15	18
8–11 (%)	9	16
12+ (%)	9	4
Average hours spent per week on HIV self-study		
0 (%)	8	4
1–2 (%)	66	62
2–4 (%)	15	23
4–7 (%)	9	6
≥8 (%)	2	6

**Figure 3:**
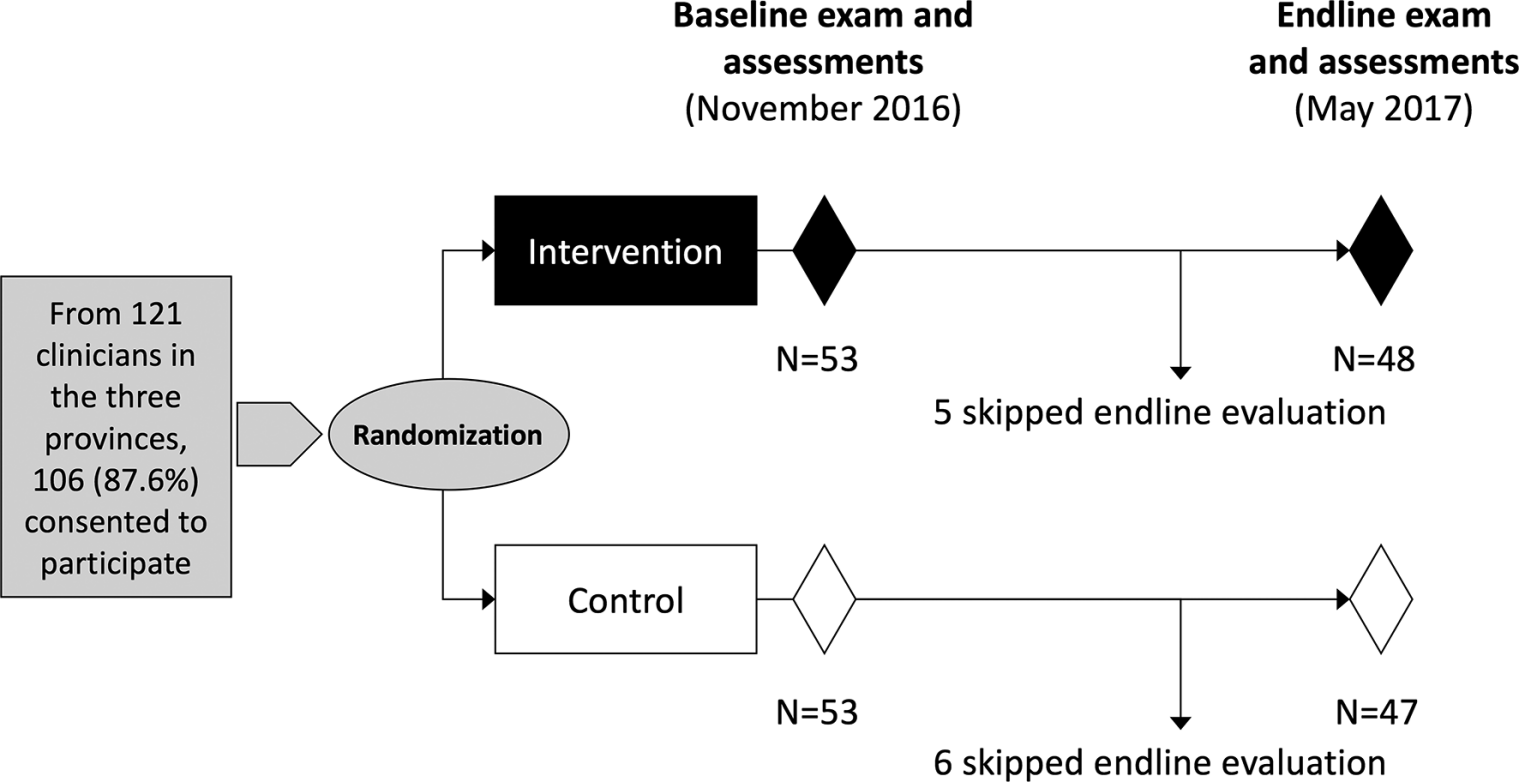
Participant flow diagram within the mobile continuing medical education study. The figure depicts the randomisation into intervention/control groups, the timing of baseline/endline assessments, the duration of the study and study attrition affecting the proportion that returned for the endline assessments.

The baseline and endline workshop were conducted in November 2016 and May 2017, respectively. As summarised in figure 3, the 106 HIV clinicians who enrolled and completed all baseline procedures were randomised 53 and 53 to the two groups. No clinicians were excluded for failing to meet eligibility criteria. Subsequently, 48 (90.6%) and 47 (88.7%) of intervention and control participants, respectively, participated in the endline workshops, with no significant difference between the two groups. The reason for non-attendance at endline was in all cases because the participant had scheduling conflicts. No participants withdrew consent.

### Impact of mCME on primary endpoint

While utilisation of the HMU courses was far from universal in either group, compared with control participants, those in the intervention were significantly more likely to ever access the HMU courses (60.4% vs 26.4%, respectively, RR 2.3, 95% CI 1.4 to 3.8). Similarly, intervention participants accessed the HMU courses a total of 134 times versus 27 times for controls, accounting for 83% of the total course utilisation across both groups (P<0.001). Though we lacked a direct measure of the amount of time users spent on the HMU web pages, a reasonable proxy for this was the total number of times participants ‘clicked’ to access different web pages or documents within each course, and this was also much higher among intervention than control participants (an average of 36.1 vs 15.3 web page clicks per participant, P=0.07). However, when limiting this analysis to participants who actually used the HMU courses, reducing the floor effect from the ‘zero click’ participants, the mean click frequency between the intervention and control groups proved to be nearly identical (59.7 vs 57.8 clicks, respectively, P=0.9). This suggests that mCME increased the proportion of intervention participants than controls who used the HMU courses, but did not change the intensity of use among those who chose to use the courses.

### Impact of mCME on secondary endpoints

**Figure 4:**
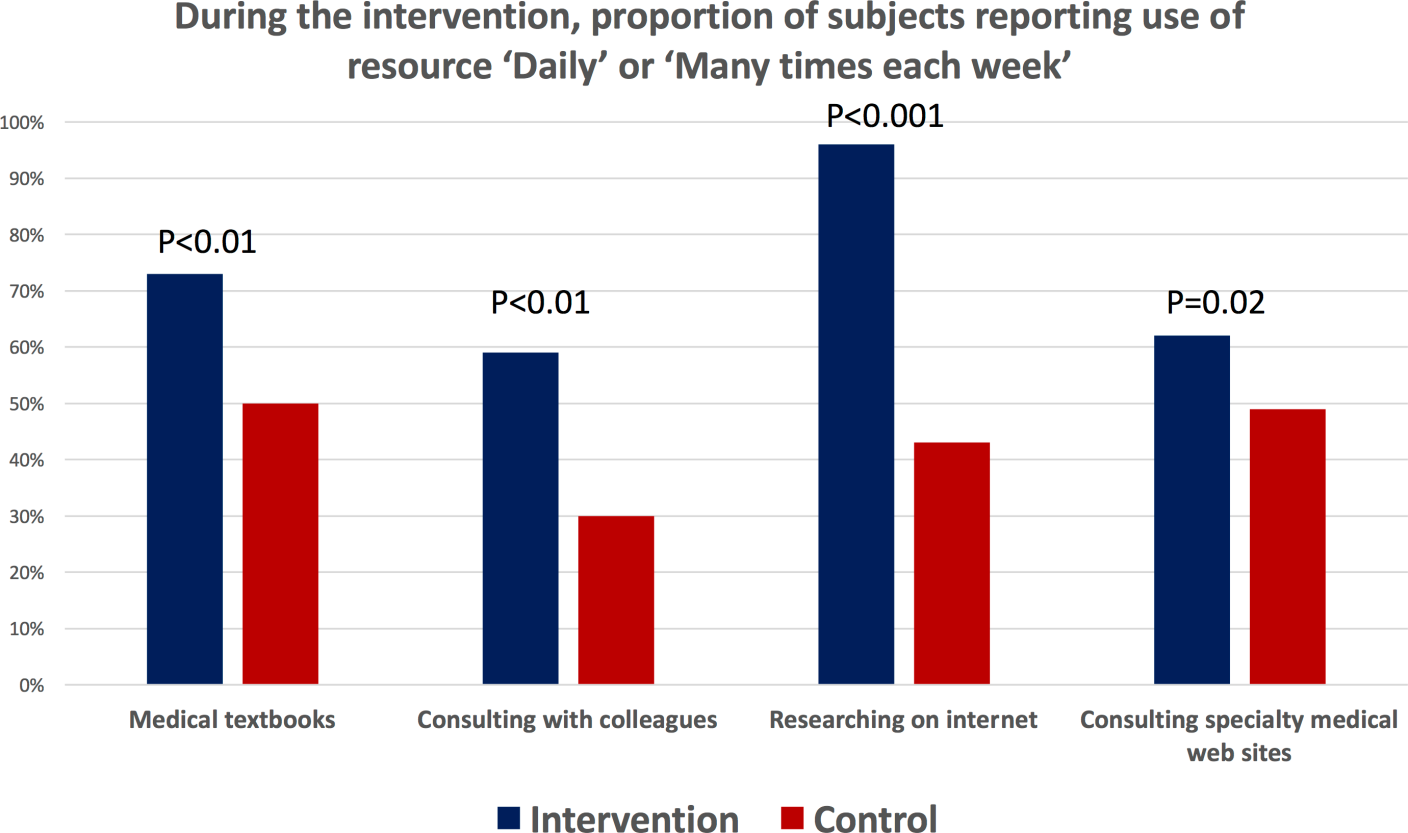
Impact of the mobile continuing medical education intervention on the frequency of self-study during the trial. The figure compares the frequency that intervention and control participants reported ‘Daily’ or ‘Many times per week’ use of each self-study modality.

**Figure 5:**
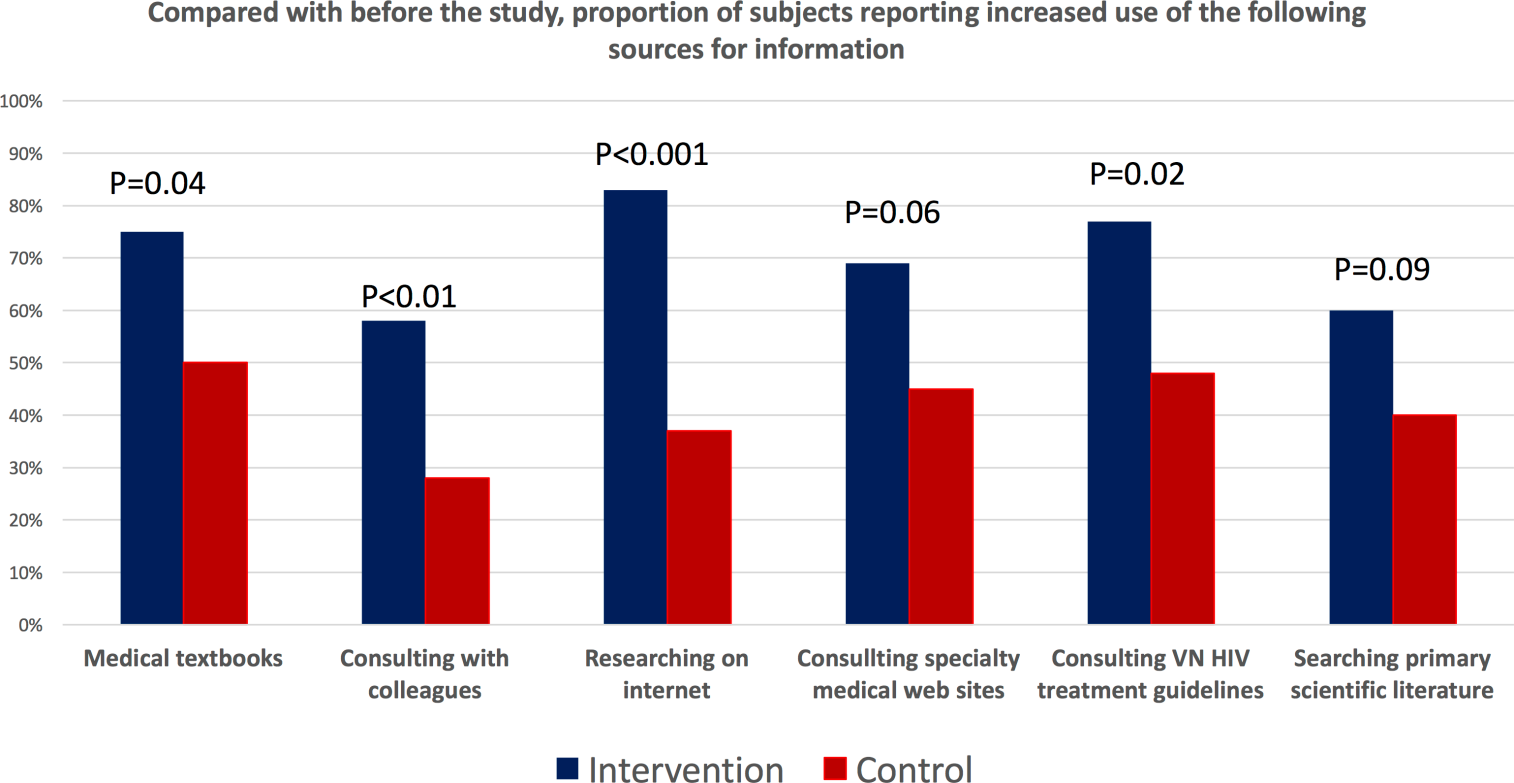
Impact of the mobile continuing medical education intervention on reported changes in self-study behaviours. The figure summarises the proportion of intervention or control participants who reported that their use of the specified self-study modalities had increased compared with prior to the study.

Self-reported measures of self-study behaviour improved among intervention participants (figure 4). They were significantly more likely than control participants to report ‘daily’ or ‘multiple times per week’ use of medical textbooks, consultation with colleagues, research on the internet and use of specialist medical websites. Similarly, in terms of a change in behaviours during the study compared with prior to the study, more intervention than control participants reported an increased use of medical textbooks, clinical consultations with colleagues, researching topics on the internet, using medical specialist websites on the internet, consulting the Vietnam HIV/AIDS treatment guidelines and searching for papers from the primary scientific medical literature (figure 5).

Among both groups, baseline exam scores were <50%. At baseline, the average scores on the medical knowledge test were somewhat lower among intervention than control participants, and this decreased the apparent magnitude of the improvement in mean test scores between the two groups (an increase of mean 10 percentage points for intervention vs an increase of mean 5 percentage points for controls). When adjusting for these baseline differences, intervention and control participants’ scores improved by +26% versus +13%, respectively, in the per-protocol analysis (P=0.06) and by +23% and +12% (P=0.05), respectively, in the ITT analysis (table 4).

**Table 4:** Impact of mobile continuing medical education on performance on medical knowledge measured as % change in mean group scores between the baseline and endline examinations

	Baseline (% correct)				Endline (% correct)				% change from baseline to endline			
	n	Mean (95% CI)	Median (IQR)	P	n	Mean (95% CI)	Median (IQR)	P	n	Mean (95% CI)	Median (IQR)	P
Per- protocol analysis												
Treatment	53	45% (41 to 48)	43% (39 to 51)	0.11	48	55% (52 to 58)	54% (49 to 61)	0.53	48	+26% (16 to 35)	+18% (7 to 42)	0.06
Control	53	48% (45 to 51)	48% (40 to 54)		47	53% (50 to 57)	54% (46 to 64)		47	+13% (5 to 22)	+9% (–4 to 33)	
Intention-to-treat analysis												
Treatment	53	45% (41 to 48)	43% (39 to 51)	0.11	53	53% (49.6 to 56.8)	53% (48 to 61)	0.9	53	23% (14.4 to 31.9)	17% (3 to 42)	0.05
Control	53	48 (45 to 51)	48 (40 to 54)		53	53% (49.3 to 56.3)	52% (45 to 63)		53	12% (4.5 to 19.4)	5% (–3 to 24)	

**Figure 6:**
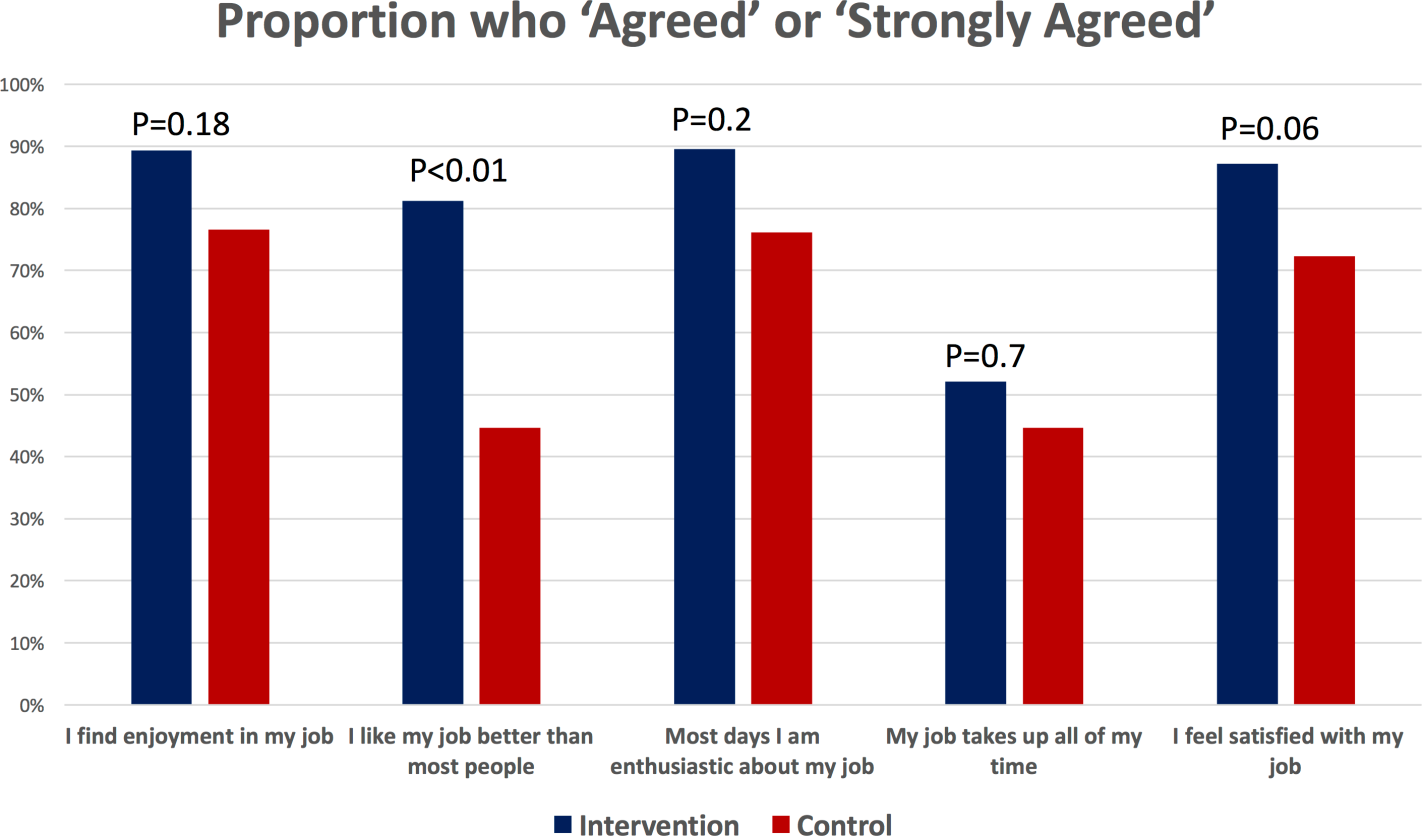
Impact of the mobile continuing medical education intervention on job satisfaction. Job satisfaction was measured at endline among intervention and control participants. The figure summarises the proportion in each group who reported that they ‘agreed’ or ‘strongly agreed’ with each of the item statements.

At endline, intervention participants reported higher job satisfaction scores across all domains (figure 6). The strongest effects were seen in the proportion of intervention versus control participants who ‘agreed’ or ‘strongly agreed’ with the job satisfaction statements, ‘I feel satisfied with my job’ (P=0.06) and ‘I like my job better than most people’ (P<0.01).

## Discussion

The revised mCME V.2.0 led to a substantial increase in medical self-study behaviours among Vietnamese HIV specialists, as reported subjectively by participants and as measured objectively around the proportion who used the online HMU CME courses. Moreover, these changes in behaviour aligned with absolute and relative gains in performance among intervention participants on a standardised medical examination, and with improved job satisfaction. We conclude that the revised mCME V.2.0 intervention was an effective yet simple strategy for motivating clinicians to engage in CME and for improving their medical knowledge.

The past decade has witnessed an explosion in the number of projects that use mobile technology to promote public health. While many mHealth educational support programmes have been reported in the literature, these tend to be small pilot projects that are underpowered or lack a control group, therefore precluding judgements about their effectiveness.^6–10^ In general, these fall into several categories: (1) Interventions directed at patients/clients that aim to effect behavioural changes or improve disease self-management and adherence^11–14^; (2) Interventions to promote clinic attendance that may be directed at clinic staff or at the clients themselves^15–17^; (3) interventions providing some sort of decision or diagnostic support using a mobile device^18 19^ and (4) efforts to provide training support to health workers using a mobile device (and was our focus in the mCME project), for which there is currently very little evidence of effectiveness. We could only locate three educational mHealth studies that were methodologically comparable to mCME. Two had no impact,^6 20^ while the third successful study was focused narrowly around a single clinical behaviour.^21^ Therefore, to our knowledge, the mCME project is the first mHealth intervention to address the challenge of promoting CME over a broad range of clinical topics.

A key benefit of the mCME approach is that it substantially increased utilisation of existing online CME course materials. In earlier discussions, the team at HMU that was responsible for hosting the online courses were frustrated by how few clinicians actually took their courses. These had required hundreds of person-hours for curriculum development, substantial costs for developing the web architecture to enrol, register and track users, and imposed recurring costs related to hosting the courses, upgrading courses over time and providing IT support to the system. Yet as of early 2016, just over 60 users were registered across all of Vietnam, roughly the same number of students as courses being offered (personal communication, Ms Ngoc Anh, Director of HMU’s IT group). This constitutes a poor return on investment, which our data suggest could be improved through integration with mCME.

Returning to our conceptual model (figure 1), the revised mCME V.2.0 intervention appeared successful in promoting the ‘strong pathway’ via lateral learning. This is an important distinction from other mHealth educational interventions where the educational curriculum is delivered entirely within the SMS messages themselves. This has practical implications given that the brevity of text messages (≤160 characters) imposes severe constraints on the volume and complexity of content that can realistically be delivered. For example, Chen *et al* reported that an SMS-based curriculum on the appropriate use of antibiotics for suspected viral infection significantly reduced unnecessary antibiotic prescriptions.^21^ This use case is quite typical of the majority of mHealth educational apps that are narrowly focused around a single topic or clinical behaviour. But that is a vastly simpler challenge compared with augmenting a deep understanding of complex medical information across a wide range of clinical competencies, as was our goal in the mCME project. For that, text messages alone are clearly insufficient. Rather, the goal is to promote a larger educational strategy and open-ended curriculum in which mCME is used to connect readers to content areas and to motivate self-study behaviours. Approaching CME from this perspective relieves the SMS messages from the burden of having to carry all relevant content, a completely unrealistic goal given the sheer scale of clinical medicine. Instead, mCME promotes daily self-study behaviours in ‘small doses’ that demand only a few minutes per day from users, while encouraging students to commit much larger—but intermittent—investments of their time, including but not limited to the use of online CME courses. This is a fundamentally different paradigm from most mHealth educational strategies to date.

There is strong evidence that interactive educational approaches are more effective than passive learning, which underlies the power behind the deceptively simple daily quiz questions and performance feedback messages.^22–27^ Here we took a cue from behavioural economics, and, through the daily quizzes and the performance feedback messages, sought to create a game-like atmosphere around mCME that promoted competition among users and thus interest and motivation. The gamification of learning is increasingly recognised as an effective pedagogical tool across diverse contexts,^28–31^ including in medical education.^32–35^ And while gamification is intended to be entertaining to the participants, simultaneously it promotes the more serious and ambitious goal of encouraging them to engage in more in-depth self-study outside of the game, which is where the ‘big doses’ of learning actually take place. This too is a fundamentally different concept for how an mHealth educational app could work.

Given these considerations, we emphasise that the mCME intervention was not designed as a substitute for primary medical training, but rather to support the retention (or recall) of previously acquired knowledge, and to motivate participants to search further for new information to help them learn about the topics presented in the daily quizzes. In clinical practice, reinforcement occurs naturally during the process of delivering care, particularly for very common medical problems. But for less common problems, for unusual presentations of common problems or to ensure that current practices are aligned with changing best practices, CME is indispensable.^36 37^ This is where our mCME intervention is intended to be most useful. By offering our users daily reminders of key concepts, facts and problems, it provides a daily series of reminders covering the scope and breadth of the core medical knowledge required, while highlighting current knowledge gaps or misconceptions. It did this by providing ‘cues to action’ through the daily SMS reminders. In this regard, one can view the main benefit of the mCME intervention as helping our participants to become better students.

We see mCME as a novel pedagogical approach that is pragmatic, flexible and generalisable since our intervention has no inherent links to any particular content area, user group or geography. For groups supporting the mCME programme, the only requirements are the software itself and the content that is to be broadcast. For participants, the only requirements are ownership of a smartphone and access to a mobile network. We note that global smartphone sales overtook feature phones in 2013 and now account for the overwhelming majority of new phone sales.^38^ Access to the internet through 3G/4G networks is becoming widespread, but could still be a limitation in settings where cellular network access is patchy and/or bandwidth constrained. However, this is of far less of a concern for SMS messages, which use a fraction of the bandwidth required for media files.

A downside to mCME in its current form is that text messages lack the visual appeal of many online educational software systems, such as the widely used Moodle system, which was what had been used for the HMU courses. However, an upside to the SMS approach is flexibility and speed. To note, the software we developed for mCME uses Microsoft Excel as its database, meaning that new content can be customised and delivered within hours simply by updating a spreadsheet on the server’s databases. This is an enormous advantage if the goal is to develop and quickly push out new information to a geographically dispersed population of users, such as in response to the outbreak of a novel infectious disease like Zika virus. Moreover, we were able to overcome the lack of elegant graphics, pictures or media content in the SMS messages themselves through the use of embedded hyperlinks.

We believe that strengths of the study included the high proportion of clinicians from the three provinces who enrolled, making the results highly representative and robust against selection biases, the rigorous methodology around the examination development and testing procedures, and the use of multiple metrics to assess changes in study behaviour (objectively and subjectively).

Our study also had several limitations. First, the sample size was relatively small, which precluded precise measurements of impact and argues for repeating this experience at a larger scale. Given that we began these investigations focused on primary care medicine, returning to the CBPA population as in mCME V.1.0, who number >50 000 across all of Vietnam, would be a logical next step. This would also provide an opportunity to conduct innovative implementation science around the process of scale up. Second, while HMU course rates were higher among intervention participants, absolute use never exceeded 20% per module, suggesting that further incentives to participation should be considered and/or that the content itself should be better tailored to meet user expectations. Results from the analyses of the qualitative data collected from participants in the second trial could be quite instructive in guiding such refinements. Third, earlier feedback from the CBPAs during mCME V.1.0 indicated a desire for more interactivity with the system or between users in the system. We note that distance learning often imposes a trade-off, where personal interactions with a professor are sacrificed for wider access to geographically dispersed participants. Nonetheless, it might be feasible to embed some form of chat system into mCME to allow participants to confer with a specialist moderator, or between each other, to facilitate feedback and discussion. In our view, this argues for proceeding towards mCME V.3.0, which might build off alternative platforms for content delivery such as ‘WhatsApp’ or the similar Vietnamese equivalents ‘Zalo’ and ‘Viber’, which host a wider range of media files, including photos, videos and audio files.

Further analyses are under way to disaggregate the components of mCME V.2.0 to better understand which were most influential in shifting behaviours, to understand the experiences of our participants through analysis of the qualitative data, to estimate the cost-effectiveness of mCME versus traditional CME, to determine the optimal frequency and timing for delivering the intervention, and to understand participant characteristics that predicted utilisation and outcomes. Our ultimate goal is for nationwide scale up of mCME, which would be suited for a large-scale implementation science project to identify the most efficient way of delivering mCME, ways to link mCME with formal CME and whether mCME could be used to register and track students, award CME credits and encourage compliance. Additionally, we believe it would be valuable to investigate whether additional forms of incentives could increase participation rates, reduce attrition over time and improve performance. These could include various strategies to publicly praise high-performing students, award certificates of merit or potentially even grant monetary or in-kind rewards.

In summary, mCME V.2.0 was popular with clinicians, was technically simple to deliver and yet was a powerful tool that promoted meaningful self-study behaviours and learning. Moreover, it is readily integrated with existing online distance learning platforms and can enhance the utilisation and hence return of those investments. The flexibility of mCME allows it to be adapted for use across diverse settings and user groups, making it highly generalisable. We conclude that this approach could be embedded within ongoing initiatives for distance learning in Vietnam as well as other settings.
