# Supplementary material for: mCME project V.2.0: randomised controlled trial of a revised SMS-based continuing medical education intervention among HIV clinicians in Vietnam
Source: BMJ Glob Health. 2018 Feb 26;3(1):e000632. doi: 10.1136/bmjgh-2017-000632 (PMC5841494; doi:10.1136/bmjgh-2017-000632)
Supplement: Supplementary file 1 [file bmjgh-2017-000632supp001.pdf]

The mCME-AIDS Sub-Study:

A nested sub-study within the ongoing mCME Project

A collaboration between

Boston University School of Public Health,

Hanoi Medical University,

the Vietnamese Ministry of Health

and Consulting, Researching on Community Development (CRCD), Ltd.

1. Introduction

1.1 Background and objectives

The mobile continuing medical education (mCME) Project was initiated to test the hypothesis that SMS text messaging could be used to support and improve medical knowledge among a cadre of Vietnamese Community Health Workers (CHWs), so-called Community Based Physician's Assistants (CBPAs). This protocol describes a nested sub-study, mCME-AIDS, which mirrors many of the approaches for the parent study, but focuses on HIV/AIDS clinicians as the target population as opposed to CBPAs. Both the parent study and the mCME-AIDS sub-study are being conducted in partnership with colleagues in Vietnam from the Ministry of Health (the Center for Population Research, Information and Databases (CIPRID)) and key academic institutions (the Hanoi School of Public Health, the Hanoi Medical University, Vietnam Administration for AIDS Control (VAAC)).

Given the smaller size of the sub study's budget, the scope and scale of the sub-study have been reduced in several ways (2 study arms instead of 3, total sample size 60-80 instead of ~660). Also in contrast with the parent study, where the content of the text messages cover a number of aspects of primary care medicine, the content in the mCME-AIDS sub study will be focused on aspects of HIV/AIDS clinical care, prevention, and harm reduction.

At their core, however, the two projects are very similar. Subjects in both groups undergo a baseline knowledge exam focused on HIV/AIDS. Following randomization, Group 1 subjects start to receive generic non-medical text messages, while Group 2 subjects receive a daily HIV/AIDS related message. These messages are presented in multiple-choice format, with four options for the participant to select from. A correct answer triggers a congratulatory message back to the subject from the server, while an in correct answer provides a consolation message along with the correct answer.

In addition, regardless of whether the response was correct or not, the text message sent back to the subject after they have given their response to the daily question will include an HTML hyperlink directing the subject to an on line eLearning module thematically linked to the daily question. For the past several years, the Vietnam Administration for AIDS Control (VAAC, a sub-division within the VN Ministry of Health, which is mandated to oversee HIV care and prevention activities in the country) has invested in development of on line eLearning course materials, spanning a wide range of clinically relevant topics. However, at present, utilization of these materials has been low. By linking our SMS intervention to the on line eLearning materials, the intervention messages serve both as a way of reinforcing knowledge about key concepts, and also as a means to direct participants to further self learning activities on these same topics. In this way, the intervention reinforces information, as well as providing a stimulus for more in depth self-learning.

At the end of six months of daily messages, the participants undergo a second examination. Our hypothesis is that intervention subjects will score significantly higher on the endline examination than control subjects. In addition, we hypothesize that exposure to the intervention will boost participant's use of the eLearning materials, compared with control subjects.

At the end of the intervention period, which will roughly coincide with the conclusion of the mCME parent study as well, several additional activities will occur. First, we will convene a series of focus group discussions with Group 2 participants to explore their experiences using SMS as a tool for distance learning and to understand their experience of using the eLearning materials. Second, we plan to convene a key stakeholder workshop in Vietnam to disseminate the results and insights from these activities with our colleagues and interested parties in country with a broader vision of planning for nationwide scale up and/or adaptation of these approaches in other contexts.

1.2 Specific objectives

O1: Develop an HIV/AIDS-specific mCME module, including the SMS content and exam questions, incorporating HTML hyperlinks between the SMS content and the VAAC's eLearning materials.

O2: Conduct an exploratory efficacy study assessing the impact of an SMS based CME intervention in terms of improving performance on a standardized examination, and boosting utilization of on-line eLearning materials.

O3: Through qualitative methods, assess user experiences with this novel educational modality.

O4: Convene a national stakeholder workshop to assess findings and plan for next steps, including a framework to integrate the mCME intervention into current and future CME activities, including eLearning.

1.3 Study team, key personnel

The key study members include the following individuals.

1. Dr. Christopher Gill, MD MS, Associate Professor of Global Health, BUSPH. Role: Principle Investigator
2. Dr. Lora Sabin, PhD MA, Associate Professor of Global Health, BUSPH. Role: Co-Investigator.
3. Dr. Rachael Bonawitz, MD, Assistant Professor of Global Health, BUSPH, Role: Co-Investigator.
4. Dr. Nafisa Halim, PhD, Assistant Professor of Global Health, BUSPH. Role: Co-Investigator, statistician.

5. Mr. Bao Ngoc Le, MA, MS, President and CEO of the NGO: Consulting, Researching on Community Development (CRCD). Role: Co-investigator, lead for VN field team; liaison with VN MOH and public health agencies/academic institutions.

2. Methods

2.1 Recruitment and Eligibility

Study subjects are HIV/AIDS clinicians (MDs, NPs, or Physicians Assistants) who are licensed in VN and have specialized in HIV clinical care and currently practice primarily or exclusively among patients infected with HIV.

Potential subjects will be included provided that they meet all of the following inclusion and exclusion criteria:

Inclusion	*Exclusion*
1. Licensed graduate from an accredited medical college in Vietnam	1. Unwilling to sign informed consent
2. Possesses a smart phone	2. Lives/operates in an area without cellular coverage
3. Phone able to send/receive text messages	3. Unwilling to adhere to study procedures
4. Aged ≥18 years	

2.2 Study Procedures

2.2.1 Allocation of subject IDs

Subjects will be assigned a unique study ID no. once they have signed consent. These will be provided sequentially as subjects enroll.

A key will be created to link subject names to the assigned ID numbers. At the conclusion of the study this key will be destroyed.

2.2.2 Randomization

Following the examination, subjects will be allocated to 1 of 2 groups using a process of restricted randomization designed to try and reach parity in the numbers of HIV clinicians in each group. Ideally, 50 subjects will be randomized to each group. The results of the randomization will be provided to each subject at the time of randomization. Randomization will be based on a pre-defined randomization list linked to subject IDs.

2.3 Data collection

Once the required numbers of HIV clinicians have been consented and randomized, subjects will be scheduled for their baseline assessment. This will occur centrally, either in one location or at several geographically located areas roughly in parallel (these need not occur on precisely the same day, but we aim to have these all done within the same week). Each examination booklet will be pre-printed at the top of each page with a given subject's unique study ID no. to match the booklet to the randomization schema. At this point, the study ID no. will be linked to each subject's cell phone number to allow the SMS reminders.

All participants will be enrolled in the on-line Hanoi Medical University CME courses using their study ID number as their log in user ID. In this way, we will be able to track the frequency that these courses are used by participants as a function of study group allocation.

The baseline assessment will include the following elements:

Part A. Self entered demographic information. This includes information regarding age, and sex; educational history (general education and medical education specifically); date of graduation from HIV CLINICIAN training; name of training college; when most recent re-training occurred; total # of govt. sponsored retraining sessions attended in the last 2 years; prior participation in HMU CME courses. This should require about 5 minutes to complete. Part A will only be administered at the baseline evaluation.

Part B. Self entered responses to job satisfaction and self-efficacy surveys. Each of these will require about 2-3 minutes to complete. This will be administered at the baseline and endline evaluations.

Part C. Professional knowledge assessment test. This is the examination that measures each HIV CLINICIAN's knowledge of key concepts related to their clinical training. The exam will consist of 100 items in multiple-choice format. Students will have 90 minutes to complete the exam. This will be administered at the baseline and endline evaluations.

Four versions of the test will be created, each covering similar domains of relevant knowledge based on the HIV CLINICIAN text, but using different questions. Versions

1.1 and 1.2 will include identical questions, but the order of the questions will be scrambled; Similarly versions 2.1 and 2.2 will include identical questions (and though covering the same thematic areas as version 1 will be all new questions), but the order of the questions in 2.1 and 2.2 will be scrambled. At the end of six months, when the HIV clinicians repeat the exam, each individual will use the alternate version to the one that they took initially, i.e., a HIV CLINICIAN who took version 1.1 or 1.2 at baseline will be administered 2.1 or 2.2 at endline; a HIV CLINICIAN who took version 2.1 or 2.2 at baseline will take 1.1 or 1.2 at endline.

This serves two objectives. First it helps minimize the risk of cheating during exams, since adjacent neighbors sitting in an exam hall are unlikely to be taking the same version of the exam as a given tester (so copying answers will not be possible). Second, it reduces the possibility that improvements on the test are simply because they remembered questions from the previous testing date.

Additional strategies to reduce the risk of cheating are:

1) All subjects must present an official photo ID in order to receive their test book (e.g., driver's license, national ID card, passport, or other photo-containing ID document)
2) The examinations will be proctored.
3) Seating for the examination will be based on an assigned seating grid arranged so that no subjects sit adjacent to any others who are taking the same examination at that time (see figure).

Legend: E1V1 = examination 1, version 1; E1V2 = examination 1, version 2; E2V1 = examination 2, version 1; E2V2 = examination 2, version 2

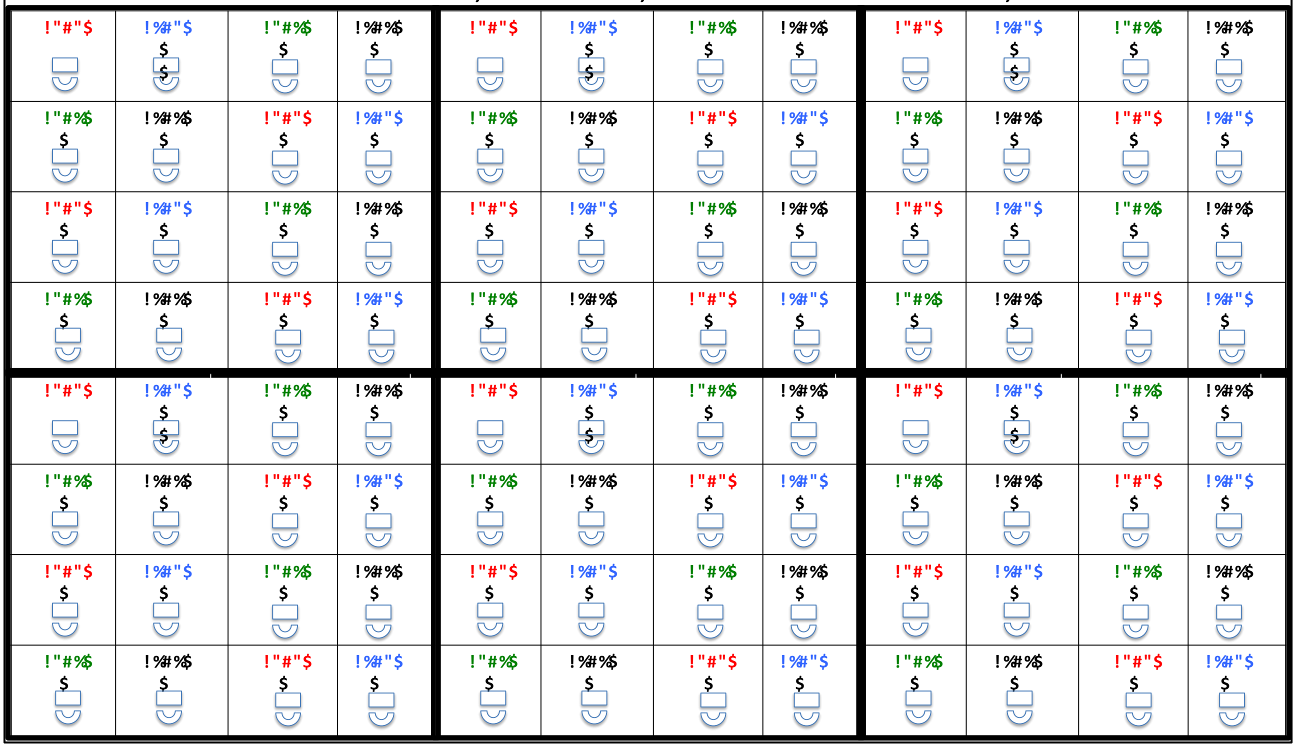

Scores in the exam will be converted to a simple numerical score on a 0-100 scale; means, medians and modes will be calculated.

2.4 The intervention

Following the baseline evaluation, HIV clinicians will return and resume their regular professional activities. Depending on their randomization group, subjects will either receive no SMS reminders (Group 1 – controls); or will receive a daily multiple-choice question, (Group 2 - intervention, interactive). As noted above, these messages will consist of a question with four answers that they must select from. Once the participant has keyed in their answer, the server is automated to inform the Group 2 subject with their answer was correct or not, and in either case to provide a hyperlink to a relevant eLearning module.

For group 2 subjects, the SMS reminders will typically be short enough to nest within the size limits of a single message. However, longer messages can be delivered over 2 or more consecutive SMS messages if needed.

At the end of six months, or specifically after 180 days have elapsed, allowing for 180 text reminders to be sent, the second assessment will be convened. Again, subjects will present to take this assessment as a proctored examination as described above. As opposed to the baseline evaluation, baseline demographic data is not collected, but only items B and C as described above.

For intervention subjects only, the text messages will be organized into a series of thematic modules, each lasting between 1 and 3 weeks of once daily messages. For example, a module could be 'HIV and tuberculosis', with all of the questions pertaining to that general area. On the first day of the module, the subjects will receive an SMS announcing the start of the module and providing a link to an on-line course hosted by Hanoi Medical University that provides CME on the same topic. This is to encourage participants to use the HMU courses. On subsequent days of the module, the terminal response SMS providing the correct answer to the daily question will also include an HTML hyperlink directing the user to technical materials pertaining to the same topic as the daily question. At the end of the module, the participants will again receive a link to the HMU course. On the Monday after completion of a module, participants will also receive an SMS providing them with the % of correct answers to the daily questions that were entered during the module, as well as the average for all of the participants on that module. Skipped questions will be treated as incorrect answers for the purposes of this calculation.

Lastly, the on-line HMU courses also host a built-in quiz. HMU will provide statistics on the proportion of subjects in both groups who use the courses, the proportion who take the quizzes, and the quiz score results, linked to participant ID number. In this way we will be able to study utilization patterns of the on line CME materials as a function of exposure to the SMS intervention.

The overall goals of the intervention are therefore to: 1) Encourage utilization of the HMU courses; 2) use SMS questions to encourage further self study; 3) use performance feedback as an additional motivator to self-study.

2.5 Development of SMS content and exam content

These will be based in part on VAAC curricula and/or other relevant sources used for training HIV clinicians, such as the VN HIV/AIDS treatment guidelines documents. Key thematic areas for focus will be identified by the key study team members, in consultation with the VN MOH and/or public health agencies. Daily SMS reminders and MCQs will be developed jointly by students and/or researchers at Boston University School of Public Health and public health students in VN. Translation will be conducted into Vietnamese by local collaborators with a final validation to ensure that the meaning of the original English versions has been retained.

2.6 Qualitative investigations

Following the second assessments, individuals up to 24 group 2 participants will be invited participate in focus group discussions, to be held the same day as the endline tests. These will be recorded and translated into English for analysis.

These subjects will be provided lunch.

The FGDs will assess the following general areas:

1). HIV clinicians attitudes regarding continuing medical education opportunities in Vietnam

2). Pros and Cons of the current system for supporting medical knowledge

3). Their impressions/opinions/likes/dislikes regarding the daily text messages, including whether they found them useful, intrusive, annoying, and whether the information that was presented was appropriate in content, format, relevance and complexity.

At this point, the data collection phase of the study is complete.

2.7 Ethical approvals/oversight

The sub-study will be approved prior to study start by the IRBs at BU and the Hanoi School of Public Health. The protocol will be registered in advance of first subject on ClinicalTrials.gov.

3. Analysis

3.1 Analytic methods

All subject data, linked to study ID Nos., will be collected on paper case report forms. Section A data will be converted to digital format through dual data entry, with reconciliation against the original paper source document between the two data entries. Section B and C data will be entered on bubble sheets and will either be entered manually via dual data entry, or automatically by digitally scanning the sheets.

Results from sections B and C will be converted to raw scores with medians, IQRs, means and standard deviations. Comparisons of mean scores will be made across each of the three groups using T tests and linear regression or other tests as indicated (see table above). In addition, the proportion of subjects scoring 85% or higher on section C will be compared using chi square tests and logistic regression or other tests, as indicated.

Analysis of the qualitative data will use the abstracted notes taken during the FGDs to identify common themes across the different domains included in the discussion guides. Data will be extracted and analyzed using nVivo software.

3.2 Data storage

Once primary data collection is complete (i.e., immediately after the endline assessment at six months in the study, and once all FGDs are complete), and the database has been cleaned and locked, the subject ID key will be destroyed.

Paper records will be warehoused in a secure, locked space at the study management center in Vietnam and retained for at least 5 years. Digital records will be warehoused at Boston University.

4. Study timeline

The estimated timeline for the project is summarized below. Note that this table assumes a Jan to December cycle, whereas in reality these would likely be relative times based on the actual study start date.

**Key Milestones**	**Q1**	**Q2**	**Q3**	**Q4**
Finalize protocol/tools	X			

IRB approvals received	X			
Group 2/3 SMS materials developed	X			
SMS system tested/validated	X			
Staff Training completed	X			
Baseline HIV CLINICIAN exam administered		X		
Period of intervention		X	X	
Endline HIV CLINICIAN exam administered			X	
Data analysis workshop			X	
Abstracts/Papers written				X
